# Systemic immune inflammation index and peripheral blood carbon dioxide concentration at admission predict poor prognosis in patients with severe traumatic brain injury

**DOI:** 10.3389/fimmu.2022.1034916

**Published:** 2023-01-09

**Authors:** Li Chen, Shaohuai Xia, Yi Zuo, Yinghong Lin, Xianshen Qiu, Qizuan Chen, Tianshun Feng, Xuewei Xia, Qixiang Shao, Shousen Wang

**Affiliations:** ^1^ Department of Neurosurgery, Fuzong Clinical Medical College of Fujian Medical University, Fuzhou, Fujian, China; ^2^ Department of Neurosurgery, Affiliated Hospital of Guilin Medical University, Guilin, Guangxi, China; ^3^ Department of Geriatrics, Affiliated Huai’an No.2 People’s Hospital of Xuzhou Medical University, Huai’an, Jiangsu, China; ^4^ Department of Neurosurgery, 900th Hospital, Fuzong Clinical Medical College of Fujian Medical University, Fuzhou, China; ^5^ Department of Neurosurgery, Ganzhou People's Hospital, No.16 Meiguan Avenue, Zhanggong District, Ganzhou, Jiangxi, China; ^6^ Institute of Medical Genetics and Reproductive Immunity, School of Medical Science and Laboratory Medicine, Jiangsu College of Nursing, No.2 the Yellow River West Road Huai'an, Jiangsu, China

**Keywords:** traumatic brain injury, system immune inflammation index, Glasgow Outcome Scale, prognosis, nomogram, carbon dioxide

## Abstract

**Background:**

Recent studies have shown that systemic inflammation responses and hyperventilation are associated with poor outcomes in patients with severe traumatic brain injury (TBI). The aim of this retrospective study was to investigate the relationships between the systemic immune inflammation index (SII = platelet × neutrophil/lymphocyte) and peripheral blood CO_2_ concentration at admission with the Glasgow Outcome Score (GOS) at 6 months after discharge in patients with severe TBI.

**Methods:**

We retrospectively analyzed the clinical data for 1266 patients with severe TBI at three large medical centers from January 2016 to December 2021, and recorded the GOS 6 months after discharge. The receiver operating characteristic (ROC) curve was used to determine the best cutoff values for SII, CO_2_, neutrophil to lymphocyte ratio (NLR), platelet to lymphocyte ratio (PLR), and lymphocyte to monocyte ratio (LMR), and chi-square tests were used to evaluate the relationships among SII, CO_2_ and the basic clinical characteristics of patients with TBI. Multivariate logistic regression analysis was used to determine the independent prognostic factors for GOS in patients with severe TBI. Finally, ROC curve, nomogram, calibration curve and decision curve analyses were used to evaluate the value of SII and coSII-CO2 in predicting the prognosis of patients with severe TBI. And we used the multifactor regression analysis method to build the CRASH model and the IMPACT model. The CRASH model included age, GCS score (GCS, Glasgow Coma Scale) and Pupillary reflex to light: one, both, none. The IMPACT model includes age, motor score and Pupillary reflex to light: one, both, none.

**Results:**

The ROC curves indicated that the best cutoff values of SII, CO_2_, PLR, NLR and LMR were 2651.43×10^9^, 22.15mmol/L, 190.98×10^9^, 9.66×10^9^ and 1.5×10^9^, respectively. The GOS at 6 months after discharge of patients with high SII and low CO_2_ were significantly poorer than those with low SII and high CO_2_. Multivariate logistic regression analysis revealed that age, systolic blood pressure (SBP), pupil size, subarachnoid hemorrhage (SAH), SII, PLR, serum potassium concentration [K^+^], serum calcium concentration [Ca^2+^], international normalized ratio (INR), C-reactive protein (CRP) and co-systemic immune inflammation index combined with carbon dioxide (coSII-CO_2_) (P < 0.001) were independent prognostic factors for GOS in patients with severe TBI. In the training group, the C-index was 0.837 with SII and 0.860 with coSII-CO_2_. In the external validation group, the C-index was 0.907 with SII and 0.916 with coSII-CO_2_. Decision curve analysis confirmed a superior net clinical benefit with coSII-CO_2_ rather than SII in most cases. Furthermore, the calibration curve for the probability of GOS 6 months after discharge showed better agreement with the observed results when based on the coSII-CO_2_ rather than the SII nomogram. According to machine learning, coSII-CO_2_ ranked first in importance and was followed by pupil size, then SII.

**Conclusions:**

SII and CO_2_ have better predictive performance than NLR, PLR and LMR. SII and CO_2_ can be used as new, accurate and objective clinical predictors, and coSII-CO_2_, based on combining SII with CO_2_, can be used to improve the accuracy of GOS prediction in patients with TBI 6 months after discharge.

## Introduction

Traumatic brain injury (TBI) refers to multiple commonly occurring physical brain injuries caused by mechanical forces (1–3). The incidence of TBI has gradually increased; by 2020, TBI became a major health problem and cause of disability (4). China has the largest number of patients with TBI worldwide, with a population-based TBI mortality rate of approximately 13/100,000, in agreement with the mortality rates reported in other countries. Acute primary TBI is characterized by destruction of brain tissue due to focal intracranial hemorrhage, epidural hematoma, subdural hematoma, cerebral contusion and diffuse axonal injury. This primary brain injury results in neuronal damage, excitatory toxicity, free radical production and inflammatory responses. These events can trigger secondary brain damage, which starts within minutes of the initial injury and often lasts for months or even years (5). Clinical treatment methods for patients with TBI are very limited, and traditional decompressive craniectomy is not adequate for all emergencies. The lack of treatment measures is due to the variety of injury types, and insufficient recognition and poor understanding of the mechanisms of secondary brain injury (6–8). TBI is a heterogeneous disease in terms of etiology, pathology, severity and prognosis, thus resulting in substantial uncertainty in the expected outcomes for individual patients (9). Therefore, methods to predict the prognosis of patients with TBI are urgently needed. Prognostic models can be used to predict outcomes according to the characteristics of individual patients. Reliable prediction results can provide information allowing physicians to perform clinical interventions more effectively and improve patient prognosis.

Clinical evidence and experiments have shown that TBI can lead to rapid inflammatory response and to secondary injury characterized by the activation of resident cells, migration and recruitment of peripheral neutrophils, and release of inflammatory mediators (10, 11). Neutrophils are the most abundant cells circulating after TBI, in both the acute phase and the post-injury period. The absolute number and frequency of circulating neutrophils is significantly higher in patients with TBI than healthy controls, and a doubling of neutrophils is observed 3 – 4.5 h after TBI (12). After neutrophils enter the brain, they release many inflammatory cytokines and factors, such as chemokines, reactive oxygen species, tumor necrosis factor (TNF-α), transforming growth factor-β (TGF-β) and interleukin-1 β (IL-1β), which in turn promote the downstream activation of intracellular signaling cascades, involving NF-Kb. Released cytokines in turn can recruit additional blood-borne neutrophils and monocytes into the injured tissue, propagating the inflammatory cascade (13). Recent clinical research has examined the ability of inflammatory biomarkers, such as the NLR, PLR and LMR, to predict outcomes after TBI. Inflammatory biomarkers after TBI have been demonstrated to have good predictive function (14–16). However, inflammation-based biomarkers are calculated on the basis of two immune cell types and do not fully indicate the role of inflammation in TBI. The SII, based on peripheral blood platelets, lymphocytes and neutrophils, more comprehensively reflects the immune state and the dynamic balance between host inflammatory process. Consequently, it has been found to provide better, more comprehensive predictions of prognosis in many types of cancer (17–19). However, the SII has not been validated in patients with TBI.

Previous studies have shown that patients with severe TBI may experience hyperventilation at early stages, thus decreasing peripheral blood CO_2_ levels. The decreases in peripheral blood CO_2_ concentrations and partial pressure of CO_2_ lead to intracranial vasoconstriction, decreased cerebral blood flow and decreased intracranial pressure (ICP), thus further aggravating the occurrence of brain metabolic disorders and inflammation after TBI (20–22). Peripheral blood CO_2_ concentrations have been found to influence the development of inflammation in mammalian innate immune and inflammatory experiments: elevated CO_2_ concentrations eliminate the NF-Kb activity induced by LPS in a manner independent of the IKKβ pathway. Hypercapnia inhibited the expression of TNF-α in macrophages stimulated by LPS and further attenuated macrophage phagocytosis. In addition, it inhibited IL-6 expression mediated by activation of multiple TLRS, while did not affect LPS-induced IL-10 or IFN-β expression. It also inhibited IL-6 expression by reducing luciferase activity driven by the IL-6 promoter. However, this process does not affect the stability of IL-6 Mrna. Hypercapnia significantly inhibits inflammation and decreases the concentration of CO_2_, thus enhancing NF-Kb activity and promoting inflammation. The use of humidified CO_2_ in laparoscopic tumor surgery decreases mesenchymal cell damage and inflammation, thereby diminishing the risk of peritoneal metastasis (23, 24). Induced hypocapnia has been used to treat elevated ICP, which is common in patients with TBI. Despite these findings, hypocapnia remains associated with adverse clinical outcomes of various forms of brain injury. Because the inhibition of cerebral blood flow may exacerbate ischemia and inflammation during acute brain injury and even lead to irreversible brain tissue infarction, chronic hypocapnia increases the risk of death and severe disability in patients with TBI (25, 26). To our knowledge, the relationship between changes in peripheral blood CO_2_ concentrations and the clinical prognosis of patients with severe TBI has not been studied. Peripheral blood CO2 concentration may be an important factor affecting inflammatory response in patients with TBI.

The objectives of this study were as follows. First, we aimed to collect data on systemic inflammatory indicators (SII, PLR, NLR and LMR), peripheral blood CO_2_ concentrations and other basic clinical characteristics of patients with severe TBI at admission. Second, we aimed to compare and analyze the predictive performance, best cutoff value and prognostic value of SII, PLR, NLR, LMR, CO_2_ and coSII-CO_2_. Third, we warranted to analyze the roles of SII and coSII-CO_2_ in predicting the prognosis of patients with TBI and to assess their order of importance by using nomograms and machine learning, to provide a valuable predictive model for physicians.

## Materials and methods

### Patient section

The study was approved by the Fuzhou 900^th^ Hospital of PLA ethics committee and conducted in accordance with the Declaration of Helsinki. As it was a retrospective study, the ethics committee approved the waiver of signed informed consent, in accordance with Chinese laws and institutional requirements. Data on patients with TBI at three large medical centers in different provinces of China from January 2016 to December 2021 were collected. The inclusion criteria were as follows: (1) types of injuries in patients with severe TBI: primary TBI caused by car accidents, high fall injury and external object strikes; (2) admission to the hospital within 12 h after injury, and completion of basic laboratory tests for basic blood indicators in the emergency department; (3) Glasgow Coma Scale score ≤ 8 at admission. The exclusion criteria were as follows: (1) death on admission; (2) clinical evidence of hematological malignancy, chronic inflammatory disease or acute infection; (3) admission to our hospital after emergency surgery in other hospitals.

### Data collection and definition

Clinical data were collected by neurosurgeons from 3 different hospitals in their own hospitals. Patients with severe craniocerebral injury were first screened out through medical records, and relevant examination and blood routine results were collected. Subsequently, GOS scores of patients for 6 months were collected through telephone, outpatient and other following-up methods. Among them, 89 patients were lost to following-up and 14 patients refused to following-up. The examined patient characteristics included age, sex, systolic pressure (SBP), pupil size, subarachnoid hemorrhage (SAH), Glasgow Coma Scale (GCS) score at admission, C-reactive protein (CRP), platelet count (PLT), platelets × neutrophils/lymphocytes (SII), neutrophil/lymphocyte ratio (NLR), platelet/lymphocyte ratio (PLR), lymphocyte/monocyte ratio (LMR),International Normalized Ratio (INR), plasma D-dimer (D-dimer), blood glucose (Glu) level at admission, Albumin (Alb), venous blood carbon dioxide (CO_2_) concentration at admission, creatine kinase (CK), creatinine (Cr), blood electrolytes, routine blood examination and length of hospital stay (LOS).

### Follow up

All patients were regularly followed up by professional staffs through outpatient service, SMS, and telephone, and discharge Glasgow Outcome Scale (GOS) assessment results were determined. Under this rating system, a GOS score of 1 indicates death, 2 indicates a persistent vegetative state, 3 indicated severe disability (conscious but disabled), 4 indicated moderate disability (disabled but independent), and 5 indicated excellent recovery with a return to baseline functional status. Dichotomized as favorable (GOS 4-5) vs. unfavorable (GOS 1-3) (27).

### Statistical analysis

SPSS21.0 (SPSS Inc., Chicago, IL) was used for data analysis. R Studio (version 4.2.0) was used for nomograms, calibration curves, decision curve analysis (DCA) and C-index calculation. GraphPad Prism 8.0.2 was used to plot bar charts and the receiver operating characteristic (ROC) curves of six indicators. Kolmogorov-Smirnov tests were used to test the normality of the data. Mean ± standard deviation was used to represent the measurement data conforming to a normal distribution, and t-tests were used for inter-group comparisons. Median and quartiles were used to represent the measurement data conforming to a normal distribution, and rank-sum tests were used for inter-group comparisons. Statistical data are expressed as percentage N (%), and comparisons between groups were performed with chi-square or Fisher’s tests. The ROC curve was used to calculate the best cutoff values for SII, CO_2_, NLR, PLR and LMR, with end points based on the patients’ GOS scores 6 months after discharge. Binary logistic analysis was performed to evaluate independent predictors of poor outcome in patients with severe TBI for factors that were significant in univariate analysis. All tests were bidirectional, and P < 0.05 was considered to indicate significant differences. Factors with P < 0.05 in univariate analysis were included in multivariate analysis. Two-sided tests were used for all statistics, and P < 0.05 was considered statistically significant. For missing data, the direct deletion method is used.

## Results

### Patient characteristics

**Figure 1:**
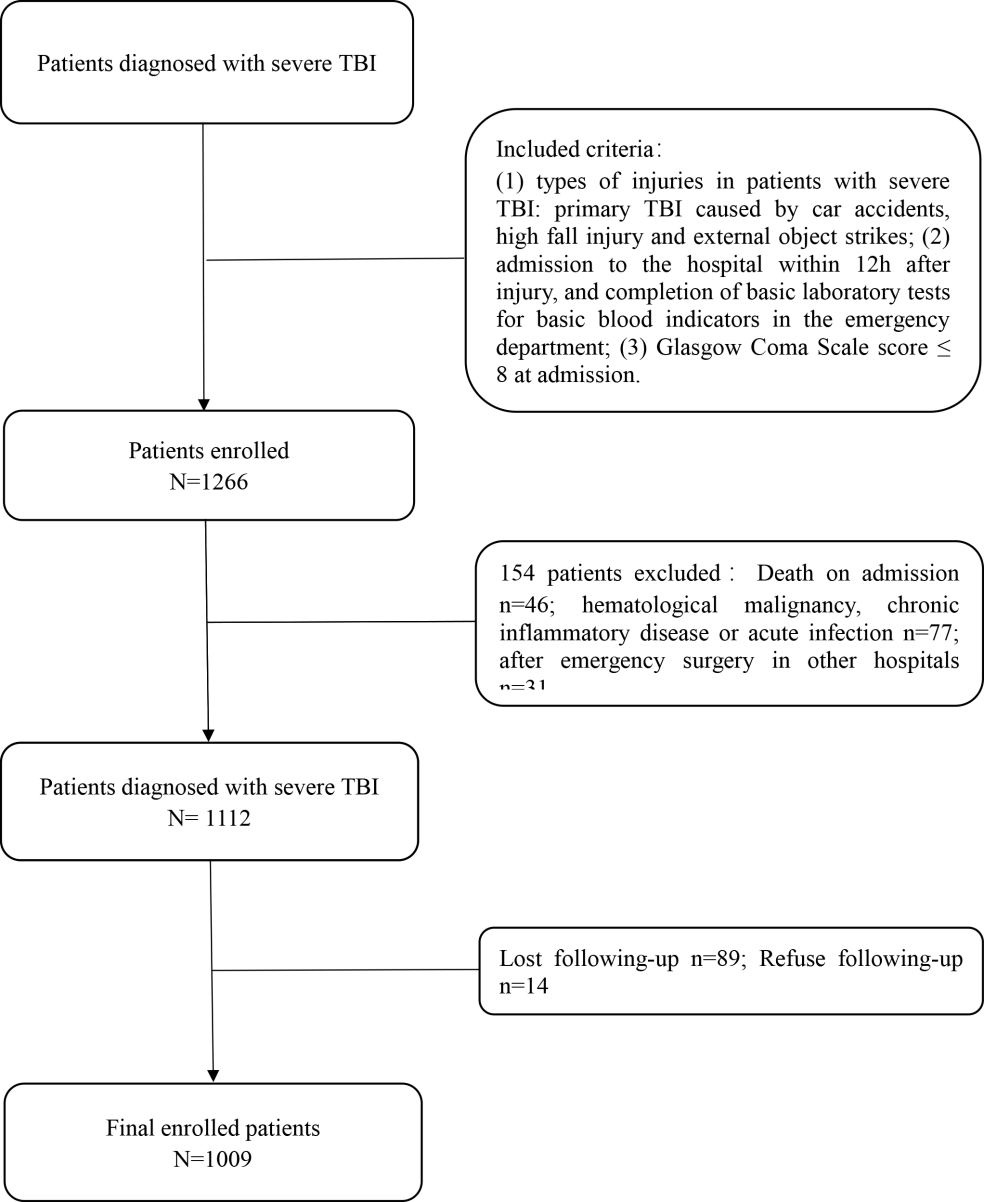
Flow chart for patients’ selection.

A total of 1266 patients with severe TBI in three large hospitals were collected from January 2016 to December 2021. Among them, 1009 patients were enrolled in the study (802 in the Fuzhou 900th Hospital of PLA, 104 in the Affiliated Hospital of Guilin Medical University and 103 in Ganzhou People’s Hospital of Jiangxi Province). A total of 802 patients from the Fuzhou 900th Hospital of PLA were included in the training group, and 207 patients from the two other centers were included in the external validation group. **Figure 1** shows the exclusion process. 1009 patients met the analysis conditions, including 223 women (22.1%) and 786 men (77.1%). The age (and interquartile range [IQR]) of the cohort was 49 [34–62] years. All 1009 patients were followed up, and the median GOS [IQR] score was 3 [1–5] at 6 months after discharge; 368 deaths (36.4%) and 641 survivors (63.6%) were recorded.

### Determination of the best cutoff values for SII, CO_2_, PLR, NLR and LMR at admission, and definition of coSII-CO_2_ grouping

The best cutoff values for SII, CO_2_, PLR, NLR and LMR in the study population were determined by ROC analysis, and GOS scores were predicted 6 months after discharge in patients with severe TBI. The GOS area under the curve (AUC) was as follows: SII=0.676 (95% CI: 0.644 - 0.710), CO_2_ = 0.648 (95% CI: 0.612 - 0.680), PLR=0.636 (95% CI: 0.6601 - 0.670), NLR=0.617 (95% CI: 0.583 - 0.653) and LMR=0.623 (95% CI: 0.586 - 0.655), corresponding to the best cutoff values of 2651.43, 22.15, 190.98, 9.66 and 1.5, respectively. According to the best cutoff values, patients were divided into two groups for analysis: a high SII group (SII ≥ 2651.43, n=336) and low SII group (SII < 2651.43, n=667); high CO_2_ group (CO_2_ ≥ 22.15, n=655) and low CO_2_ group (CO_2_ < 22.15, n=347); high PLR group (PLR ≥ 190.98, n=357) and low PLR group (PLR < 190.98, n=646); high NLR group (NLR ≥ 9.66, n=551) and low NLR group (NLR < 9.66, n=452); and high LMR group (LMR ≥ 1.5, n=629) and low LMR group (LMR < 1.5, n=374). coSII-CO_2_ groups were defined. Patients with high SII and low CO_2_ were assigned 3 points, patients with high SII and high CO_2_ were assigned 2 points, patients with low SII and low CO_2_ were assigned 1 point, and patients with low SII and high CO_2_ were assigned 0 points.

### Relationships among clinical characteristics of patients with severe TBI and SII, CO_2_, PLR, NLR, LMR

SII correlated with SBP, pupil size, SAH, Glu, [K^+^], INR, CRP, NEU, LYM and MON (P < 0.05), but not with sex, age, LOS, Cr, Alb, CK, serum sodium concentration [Na^+^], [Ca^2+^], D-dimer and PLT (P > 0.05). CO_2_ was associated with LOS, pupil size, Alb, [Ca^2+^] and INR (P < 0.05). Sex, age, SBP, SAH, Cr, Glu, CK, [K^+^], [Na^+^], [Ca^2+^], D-dimer, CRP, NEU, PLT, LYM and MON were not correlated (P > 0.05). PLR correlated with SBP, pupil size, Glu, [Na^+^] and LYM (P < 0.05), but not sex, age, LOS, SAH, Cr, Alb, CK, [K^+^], [Ca^2+^], D-dimer, INR, CRP, NEU, PLT and MON (P > 0.05). NLR was associated with SBP, pupil size, Glu, CK, INR, NEU and LYM (P < 0.05), but not sex, age, LOS, SAH, Cr, Alb, [K^+^], [Na^+^], [Ca^2+^], D-dimer, CRP, PLT and MON (P > 0.05). LMR was correlated with sex, SBP, pupil size, Glu, CK, D-dimer, INR, CRP, NEU, LYM and MON (P < 0.05), but not with age, LOS, SAH, Cr, Alb, [K^+^], [Na^+^], [Ca^2+^] and PLT (P > 0.05; **Table 1**).

**Table 1:** Relationships among SII, CO_2_, PLR, NLR, and LMR, and basic clinical features in patients with severe TBI.

Variables	SII			CO_2_			PLR			NLR			LMR		
	High (≥2651.43)	Low (<2651.43)	*P*	High (≥22.15)	Low (<22.15)	*P*	High (≥190.98)	Low (<190.98)	*P*	High (≥9.66)	Low (<9.66)	*P*	High (≥1.5)	Low (<1.5)	*P*
	n=336	n=667		n=655	n=347		n=357	n=646		n=551	n=452		n=629	n=374	
Gender			0.573			0.094			0.876			0.356			0.030
Man	258	523		521	260		277	504		423	358		476	305	
Female	78	144		134	87		80	142		128	94		153	69	
Age			0.55			0.285			0.102			0.584			0.988
	50(32.25,62.75)	48(34,61)		48(33,62)	50(35,62)		50(35,63)	48(32,61)		49(34,62)	48(33,62)		48(33,62)	49.5(34,62)	
LOS			0.051			0.005			0.123			0.106			0.441
	10(2,20)	11(4,20)		12(4,20)	9(2,19)		10(3,19.5)	12(4,20)		12(4,21)	10(4,19)		11(4,19.5)	12(3,21.25)	
SBP			0.035			0.561			0.007			0.035			0.016
	134(117.25,151)	130(118,146)		130(120,148)	112(130,150)		134(120,152)	130(116.75,146)		131(119,150)	130(115.25,143)		130(115,146)	133(120,150)	
Pupil			<0.001			<0.001			<0.001			0.001			<0.001
Unequal	171	221		222	170		166	226		241	151		213	179	
Equal	165	446		433	177		191	420		310	301		416	195	
SAH			0.011			0.587			0.058			0.096			0.373
Yes	229	399		407	222		237	391		358	270		387	241	
No	107	267		247	125		119	255		193	181		241	133	
Miss		1		1			1				1		1		
Cr			0.563			0.250			0.486			0.889			0.380
Abnormal	71	131		125	76		76	126		110	92		132	70	
Normal	264	536		528	266		280	520		410	360		496	304	
Miss	1			2	5		1			1			1		
Glu			<0.001			0.061			0.040			<0.001			<0.001
Abnormal	315	575		572	313		326	564		510	380		541	349	
Normal	20	92		81	29		30	82		40	72		87	25	
Miss	1			2	5		1			1			1		
ALB			0.517			0.005			0.479			0.995			0.612
Abnormal	172	326		304	190		183	315		274	224		308	190	
Normal	163	337		348	149		173	327		275	225		317	183	
Miss	1			3	8		1	4		2	3		4	1	
CK			0.079			0.251			0.937			0.037			<0.001
Abnormal	268	513		501	273		272	509		438	343		467	314	
Normal	51	134		128	57		65	120		88	97		141	44	
Miss	17	20		26	17		20	17		25	12		21	16	
K^+^			0.01			0.132			0.709			0.062			0.060
Abnormal	106	160		162	100		97	169		159	107		154	112	
Normal	229	507		491	242		259	477		391	345		474	262	
Miss	1			2	5		1			1			1		
Na^+^			0.059			0.700			0.002			0.143			0.079
Abnormal	139	236		242	131		156	219		217	158		222	153	
Normal	196	431		411	211		200	427		333	294		406	221	
Miss	1			2	5		1			1			1		
Ca^2+^			0.201			0.006			0.403			0.410			0.409
Abnormal	121	214		199	134		125	210		190	145		204	131	
Normal	214	453		454	208		231	436		360	307		424	243	
Miss	1			2	5		1			1			1		
D-Dimer			0.103			1.000			1.000			0.051			0.050
Abnormal	326	639		632	326		345	620		531	434		603	362	
Normal	0	7		5	2		2	5		1	6		7	0	
Miss	10	21		18	19		10	21		19	12		19	12	
INR			0.001			0.001			0.412			0.010			0.002
Abnormal	116	164		162	116		105	175		172	108		154	126	
Normal	213	484		479	213		242	455		365	332		457	240	
Miss	7	19		14	18		10	16		14	12		18	8	
CRP			0.003			0.105			0.245			0.287			0.001
Abnormal	225	379		383	217		227	377		348	256		345	259	
Normal	90	236		224	100		110	216		176	150		221	105	
Miss	21	52		48	30		20	53		27	46		63	10	
NEU			<0.001			0.287			0.968			<0.001			0.004
Abnormal	333	603		614	315		333	603		546	390		576	360	
Normal	3	64		40	27		24	43		5	62		53	14	
Miss				1	5										
PLT			0.099			0.967			0.074			0.232			0.330
Abnormal	54	136		125	65		57	133		97	93		125	65	
Normal	282	531		529	277		300	513		454	359		504	309	
Miss				1	5										
LYM			<0.001			0.589			<0.001			<0.001			<0.001
Abnormal	243	228		312	157		288	183		379	92		197	274	
Normal	93	439		342	185		69	463		172	360		432	100	
Miss				1	5										
MON			0.004			0.084			0.159			0.120			<0.001
Abnormal	221	376		376	216		202	392		340	257		287	310	
Normal	115	291		278	126		155	251		211	195		342	64	
Miss				1	5										

LOS, length of hospital stay; SBP, systolic pressure; SAH, subarachnoid hemorrhage; Cr, creatinine; Glu, glucose; ALB, albumin; CK, creatine kinase; INR, International Normalized Ratio; CRP, C-reactive protein; NEU, neutrophile; LYM, lymphocyte; MON, monocyte. All data were analyzed with chi-square test, rank sum test, and Fisher’s precision probability test, and P < 0.05 was considered to indicate statistically significant differences.

### Relationships among SII, CO_2_, PLR, NLR, LMR, coSII-CO_2_ and GOS scores at 6 months after discharge in patients with severe TBI

**Figure 2:**
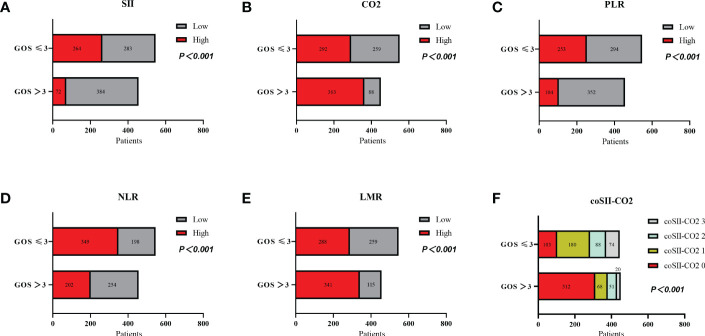
Relationships among levels of SII, CO_2_, PLR, NLR, and LMR, and coSII-CO_2_ and GOS in patients with severe TBI. **(A)** Patients with high SII ≥ 2651.43 had poorer GOS than patients with low SII<2651.43 (P < 0.001). **(B)** Patients with low CO_2_<22.15 had poorer GOS than patients with high CO_2_ ≥ 22.15 (P < 0.001). **(C)** Patients with high PLR ≥ 190.98 had poorer GOS than patients with low PLR<190.98 (P < 0.001). **(D)** Patients with high NLR ≥ 9.66 had poorer GOS than patients with low NLR<9.66 (P < 0.001). **(E)** Patients with low LMR<1.5 had poorer GOS than patients with high LMR ≥1.5 (P < 0.001). **(F)** GOS scores of patients: coSII-CO_2_ 0 > coSII-CO_2_ 1 > coSII-CO_2_ 2 > coSII-CO_2_ 3 (P < 0.001).

The GOS significantly differed from SII, CO_2_, PLR, NLR, LMR and coSII-CO_2_ in patients with severe TBI (P < 0.001). Patients with GOS > 3 tended to have low SII (84%), high CO_2_ (80%), low PLR (77%), low NLR (55%), high LMR (74%) and coSII-CO_2_ scores of 0 (69%). Patients with GOS ≤ 3 showed opposite trends (**Figure 2** and **Table 2**).

**Table 2:** Relationships among the levels of SII, CO_2_, PLR, NLR, and LMR, and the coSII-CO_2_ and GOS scores in patients with TBI.

Variables	SII			CO_2_			PLR			NLR			LMR			coSII-CO_2_				
	High (≥2651.43)	Low (<2651.43)	P	High (≥22.15)	Low (<22.15)	P	High (≥190.98)	Low (<190.98)	P	High (≥9.66)	Low (<9.66)	P	High (≥1.5)	Low (<1.5)	P	0	1	2	3	P
	n=336	n=667		n=655	n=347		n=357	n=646		n=551	n=452		n=629	n=374		n=415	n=248	n=239	n=94	
GOS			<0.001			<0.001			<0.001			<0.001			<0.001					<0.001
>3	72	384		363	88		104	352		202	254		341	115		312	68	51	20	
≤3	264	283		292	259		253	294		349	198		288	259		103	180	188	74	

SII, platelets × neutrophils/lymphocytes; NLR, neutrophil/lymphocyte ratio; PLR, platelet/lymphocyte ratio; LMR, lymphocyte/monocyte ratio; CO_2_, carbon dioxide; coSII-CO_2_, Systemic immune inflammatory index combined with carbon dioxide; GOS, Glasgow Outcome Scale. Patients with high SII and low CO2 were assigned 3 points, patients with high SII and high CO2 were assigned 2 points, patients with low SII and low CO2 were assigned 1 point, and patients with low SII and high CO2 were assigned 0 points. All data were analyzed with chi-square test, and P < 0.05 was considered to indicate statistically significant differences.

### Prognostic value of coSII-CO_2_ in patients with severe TBI

**Figure 3:**
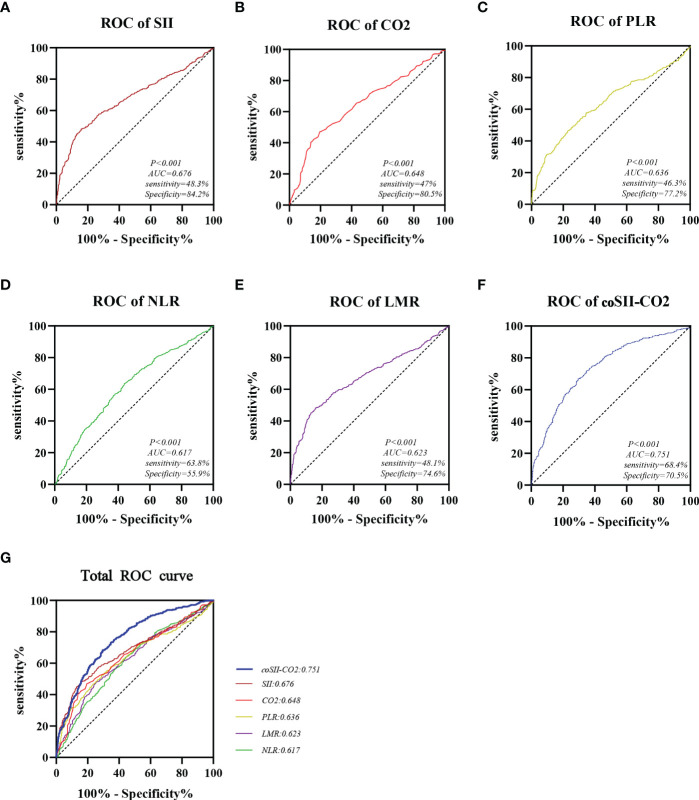
Areas under the curves of SII, CO_2_, PLR, NLR, LMR, and coSII-CO_2_. **(A)** The area under the curve of SII was 0.676. **(B)** The area under the curve of CO_2_ was 0.648. **(C)** The area under the curve of PLR was 0.636. **(D)** The area under the curve of NLR was 0.617. **(E)** The area under the curve of LMR was 0.623. **(F)** The area under the curve of coSII-CO_2_ was 0.751. **(G)** Compared with other independent predictive indicators, the area under the curve of coSII-CO_2_ in the combined group was largest, thus indicating that coSII-CO_2_ was the most accurate and reliable indicator for predicting the prognosis of patients with TBI.

The predictive accuracy of SII, CO_2_, PLR, NLR, LMR and coSII-CO_2_ for GOS 6 months after discharge were compared by ROC curve analysis. The AUC of SII, CO_2_, PLR, NLR, LMR and coSII-CO_2_ were as follows: SII=0.676 (95%CI: 0.644–0.710), CO_2_ = 0.648 (95%CI: 0.612–0.680), PLR=0.636 (95%CI: 0.601–0.670), NLR=0.617 (95%CI: 0.583–0.653), LMR=0.623 (95%CI: 0.586–0.655) and coSII-CO_2_ = 0.751 (95%CI: 0.721–0.781). The coSII-CO_2_ had a higher AUC than SII or CO_2_ alone, thus suggesting that coSII-CO_2_ was the most reliable of these predictors for GOS 6 months after discharge and could be used as a predictive tool (**Figure 3**).

### Univariate and multivariate analysis of factors influencing GOS scores in patients with severe TBI

Univariate analysis indicated that age, SBP, pupil size, SAH, CO_2_, SII, PLR, NLR, LMR, Glu, Alb, Ck, [K^+^], [Na^+^], D-dimer, INR, CRP, LYM, MON and coSII-CO_2_ were important prognostic factors affecting GOS scores in patients with severe TBI. Multivariate logistic regression analysis showed that age (OR=1.576, 95% CI: 1.036 - 2.398, P=0.034), SBP (OR=1.925, 95%CI: 1.304 2.843, P=0.001), pupil size (OR=5.373, 95% CI: 3.614 7.988, P < 0.001), SAH (OR=1.472, 95% CI: 0.558 2.357, P=0.044), SII (OR=6.701, 95% CI: 3.821 11.752, P < 0.001), PLR (OR=1.671, 95% CI: 1.038 2.689, P=0.035), [K^+^] (OR=1.884, 95% CI: 1.222 2.904, P=0.004), [Ca^2+^] (OR=2.024, 95% CI: 1.321 3.100, P=0.001), INR (OR=3.138, 95% CI: 2.032 - 4.847, P < 0.001), CRP (OR=1.828, 95% CI: 1.242 - 2.694, P=0.002) and coSII-CO_2_ (OR=8.337, 95% CI: 3.579 - 19.606, P < 0.001) were independent prognostic factors affecting GOS in patients with severe TBI (**Table 3**).

**Table 3:** Univariate and multivariate logistic regression models used to analyze the factors influencing GOS in patients with TBI .

Variable		Univariate analysis		Multivariate analysis	
	Patients	OR (95% CI)	*P*	OR (95% CI)	*P*
Gender			0.786		
Male	786	0.959 (0.711-1.294)			
Female	223	1			
Age			0.008		0.034
≥60	299	1.450 (1.101-1.909)		1.576 (1.036-2.398)	
<60	710	1		1	
SBP			<0.001		0.001
Abnormal	376	2.242 (1.719-2.924)		1.925 (1.304-2.843)	
Normal	633	1		1	
Miss					
Pupil			<0.001		<0.001
Unequal	395	6.509 (4.840-8.755)		5.373 (3.614-7.988)	
Equal	614	1		1	
SAH			0.003		0.044
Yes	633	1.466 (1.134-1.896)		1.472 (0.558-2.357)	
No	375	1		1	
Miss	1				
CO_2_			<0.001		
High (≥22.15)	655	0.273 (0.205-0.364)			
Low (<22.15)	347	1		1	
Miss	7				
SII			<0.001		<0.001
High (≥2651.43)	336	4.975 (3.677-6.732)		6.701 (3.821-11.752)	
Low (<2651.43)	667	1		1	
Miss	6				
PLR			<0.001		0.035
High (≥190.98)	357	2.913 (2.210-3.838)		1.671 (1.038-2.689)	
Low (<190.98)	646	1		1	
Miss	6				
NLR			<0.001		
High (≥9.66)	551	2.216 (1.719-2.857)			
Low (<9.66)	452	1			
Miss	6				
LMR			<0.001		
High (≥1.5)	629	0.375 (0.286-0.491)			
Low (<1.5)	374	1			
Miss	6				
Cr			0.535		
Abnormal	202	1.104 (0.809-1.506)			
Normal	800	1			
Miss	7				
Glu			<0.001		
Abnormal	890	3.967 (2.547-6.180)			
Normal	112	1			
Miss	7				
ALB			<0.001		
Abnormal	498	1.843 (1.432-2.371)			
Normal	500	1			
Miss	11				
Ck			<0.001		
Abnormal	781	1.877 (1.356-2.598)			
Normal	185	1			
Miss	43				
K^+^			<0.001		0.004
Abnormal	266	2.428 (1.800-3.275)		1.884 (1.222-2.904)	
Normal	736	1		1	
Miss	7				
Na^+^			0.005		
Abnormal	375	0.687 (0.530-0.891)			
Normal	627	1			
Miss	7				
Ca^2+^			<0.001		0.001
Abnormal	335	2.482 (1.882-3.274)		2.024 (1.321-3.100)	
Normal	667	1		1	
Miss	7				
D-Dimer			0.069		
Abnormal	965	7.159 (0.859-59.690)			
Normal	7	1			
Miss	37				
INR			<0.001		<0.001
Abnormal	280	4.441 (3.227-6.111)		3.138 (2.032-4.847)	
Normal	697	1		1	
Miss	32				
CRP			<0.001		0.002
Abnormal	604	2.006 (1.527-2.636)		1.828 (1.242-2.694)	
Normal	327	1			
Miss	78				
NEU			0.532		
Abnormal	936	0.852 (0.516-1.408)			
Normal	67	1			
Miss	6				
PLT			0.951		
Abnormal	190	1.010 (0.735-1.387)			
Normal	813	1			
Miss	6				
LYM			<0.001		
Abnormal	471	1.772 (1.377-2.280)			
Normal	532	1			
Miss	6				
MON			<0.001		
Abnormal	597	1.635 (1.268-2.108)			
Normal	406	1			
Miss	6				
coSII-CO_2_			<0.001		<0.001
0	415	1		1	
1	248	8.018 (5.611-11.458)		8.337 (3.579-19.606)	
2	239	11.166 (7.626-16.349)			
3	94	11.208 (6.519-19.268)			
Miss	13				

LOS, length of hospital stay; SBP, systolic pressure; SAH, subarachnoid hemorrhage; Cr, creatinine; Glu, glucose; ALB, albumin; CK, creatine kinase; INR, International Normalized Ratio; CRP, C-reactive protein; NEU, neutrophile; LYM, lymphocyte; MON, monocyte; SII, platelets × neutrophils/lymphocytes; NLR, neutrophil/lymphocyte ratio; PLR, platelet/lymphocyte ratio; LMR, lymphocyte/monocyte ratio; CO2, carbon dioxide; coSII-CO2, Systemic immune inflammatory index combined with carbon dioxide; GOS, Glasgow Outcome Scale. Patients with high SII and low CO2 were assigned 3 points, patients with high SII and high CO2 were assigned 2 points, patients with low SII and low CO2 were assigned 1 point, and patients with low SII and high CO2 were assigned 0 points. P< 0.05 was considered to indicate statistically significant differences.

### Comparison of basic clinical characteristic data between the training group and external validation group

The baseline characteristics of patients in the training group and the validation group were essentially the same, and no significant differences were observed between groups (P > 0.05), thus indirectly demonstrating that the clinical characteristic data in this study were not affected by the different hospitals (**Table 4**).

**Table 4:** Clinical characteristics of the training group and the external validation group.

Variables	Total	Training group	Validation group	*P*
Gender				0.561
Male	786(77.9%)	626(77.5%)	160(79.6%)	
Female	223(22.1%)	182(22.5%)	41(20.4%)	
Age				0.633
	49(34,63)	49(34,62)	50(34,63)	
LOS				0.032
	11(3,20)	11(4,20)	9(1,17)	
SBP				0.673
	130(118,150)	130(118,150)	130(118,150)	
Pupil				0.391
Unequal	395(39.1%)	311(38.5%)	84(41.8%)	
Equal	614(60.9%)	497(61.5%)	117(58.2%)	
SAH				0.079
Yes	633(62.7%)	496(61.4%)	137(68.2%)	
No	375(37.2%)	311(38.5%)	64(31.8%)	
Miss	1	1		
CO_2_				0.515
High(≥22.15)	655(64.9%)	521(64.5%)	134(66.7%)	
Low(<22.15)	347(34.4%)	282(34.9%)	65(32.3%)	
Miss	7	5	2	
SII				0.106
High(≥2651.43)	336(33.3%)	259(32.1%)	77(38.3%)	
Low(<2651.43)	667(66.1%)	543(67.2%)	124(61.7%)	
Miss	6	6		
PLR				0.569
High(≥190.98)	357(35.4%)	282(34.9%)	75(37.3%)	
Low(<190.98)	646(64.0%)	520(64.4%)	126(62.7%)	
Miss	6	6		
NLR				0.802
High(≥9.66)	551(54.6%)	439(54.3%)	112(55.7%)	
Low(<9.66)	452(44.8%)	363(44.9%)	89(44.3%)	
Miss	6	6		
LMR				0.101
High(≥1.5)	629(62.3%)	513(63.5%)	116(57.7%)	
Low(<1.5)	374(37.1%)	289(35.8%)	85(42.3%)	
Miss	6	6		
Cr				0.281
Abnormal	202(20%)	156(19.3%)	46(22.9%)	
Normal	800(79.3%)	645(79.8%)	155(77.1%)	
Miss	7	7		
Glu				0.105
Abnormal	890(88.2%)	705(87.3%)	185(92.0%)	
Normal	112(11.1%)	96(11.9%)	16(8.0%)	
Miss	7	7		
ALB				0.065
Abnormal	498(49.4%)	386(47.8%)	112(55.7%)	
Normal	500(49.6%)	411(50.9%)	89(44.3%)	
Miss	11	11		
CK				0.496
Abnormal	781(77.4%)	620(76.7%)	161(80.1%)	
Normal	185(18.3%)	151(18.7%)	34(16.9%)	
Miss	43	37	6	
K^+^				0.407
Abnormal	266(26.4%)	208(25.7%)	58(28.9%)	
Normal	736(72.9%)	593(73.4%)	143(71.1%)	
Miss	7	7		
Na^+^				0.843
Abnormal	375(37.2%)	301(37.3%)	74(36.8%)	
Normal	627(62.1%)	500(61.9%)	127(63.2%)	
Miss	7	7		
Ca^2+^				0.841
Abnormal	335(33.2%)	269(33.3%)	66(32.8%)	
Normal	667(66.1%)	532(65.8%)	135(67.2%)	
Miss	7	7		
D-Dimer				1
Abnormal	965(95.6%)	771(95.4%)	194(96.5%)	
Normal	7(0.7%)	6(0.7%)	1(0.5%)	
Miss	37	31	6	
INR				0.082
Abnormal	280(27.8%)	214(26.5%)	66(32.8%)	
Normal	697(69.1%)	567(70.2%)	130(64.7%)	
Miss	32	27	5	
CRP				0.392
Abnormal	604(59.9%)	479(59.3%)	125(62.2%)	
Normal	327(32.4%)	267(33.0%)	60(29.9%)	
Miss	78	62	16	
NEU				0.443
Abnormal	936(92.8%)	746(92.3%)	190(94.5%)	
Normal	67(6.6%)	56(6.9%)	11(5.5%)	
Miss	6	6		
PLT				0.412
Abnormal	190(18.8%)	156(19.3%)	34(16.9%)	
Normal	813(80.6%)	646(80.0%)	167(83.1%)	
Miss	6	6		
LYM				0.923
Abnormal	471(46.7%)	376(46.5%)	95(47.3%)	
Normal	532(52.7%)	426(52.7%)	106(52.7%)	
Miss	6	6		
MON				0.09
Abnormal	597(59.2%)	376(46.5%)	136(67.7%)	
Normal	406(40.2%)	426(56.7%)	65(32.3%)	
Miss	6	6		
coSII-CO_2_				0.429
0	415(41.1%)	337(41.7%)	78(38.8%)	
1	248(24.6%)	203(25.1%)	45(22.4%)	
2	239(23.7%)	183(22.6%)	56(27.9%)	
3	94(9.3%)	74(9.2%)	20(10.0%)	
Miss	13	11	2	

LOS, length of hospital stay; SBP, systolic pressure; SAH, subarachnoid hemorrhage; Cr, creatinine; Glu, glucose; ALB, albumin; CK, creatine kinase; INR, International Normalized Ratio; CRP, C-reactive protein; NEU, neutrophile; LYM, lymphocyte; MON, monocyte; SII, platelets × neutrophils/lymphocytes; NLR, neutrophil/lymphocyte ratio; PLR, platelet/lymphocyte ratio; LMR, lymphocyte/monocyte ratio; CO2, carbon dioxide; coSII-CO2, Systemic immune inflammatory index combined with carbon dioxide; GOS, Glasgow Outcome Scale. Patients with high SII and low CO2 were assigned 3 points, patients with high SII and high CO2 were assigned 2 points, patients with low SII and low CO2 were assigned 1 point, and patients with low SII and high CO2 were assigned 0 points. All data were analyzed with chi-square test, rank sum test and Fisher’s precision probability test, and P>0.05 was considered to indicate no statistical difference.

### Comparison of nomogram predictive performance based on SII and coSII-CO_2_

**Figure 4:**
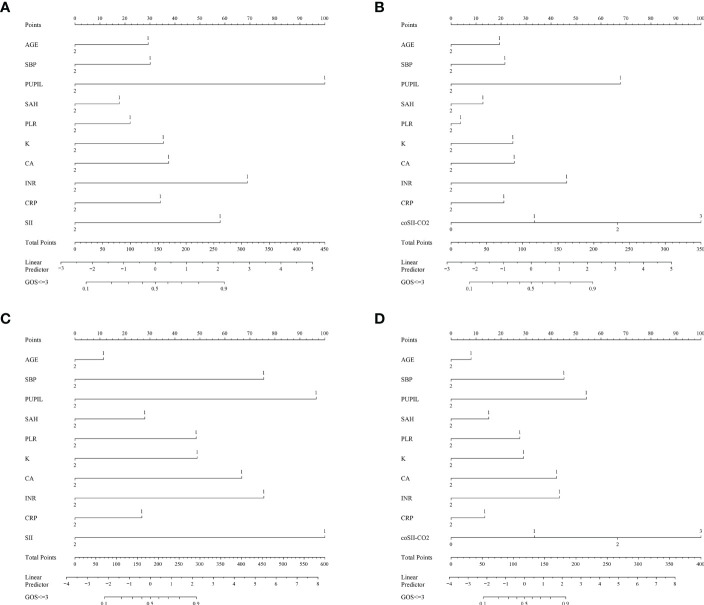
Establishment of the SII and coSII-CO_2_ nomograms in the training and validation groups. **(A, B)** Nomograms based on SII and coSII-CO_2_ in the training group. **(C, D)** Nomograms based on SII and coSII-CO_2_ in the validation group.

**Figure 5:**
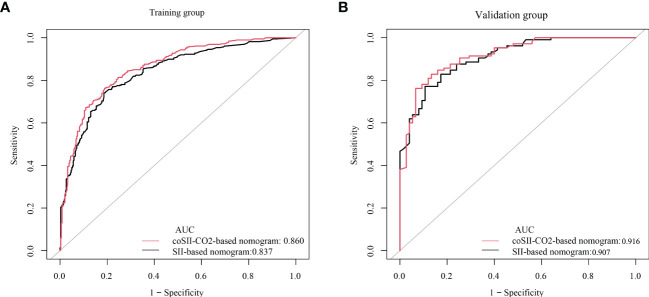
C-index based on SII and coSII-CO_2_ in the training and validation groups. **(A)** The C-index of SII and coSII-CO_2_ in the training group was 0.837 and 0.860, respectively. **(B)** The C-index of SII and coSII-CO_2_ in the validation group was 0.907 and 0.916, respectively.

**Figure 6:**
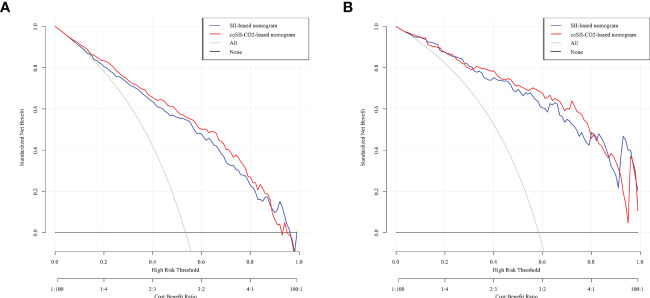
Decision curve analysis (DCA) based on SII and coSII-CO_2_ in the training and validation groups. **(A)** Clinical DCA of the training group. **(B)** Clinical DCA of the validation group.

**Figure 7:**
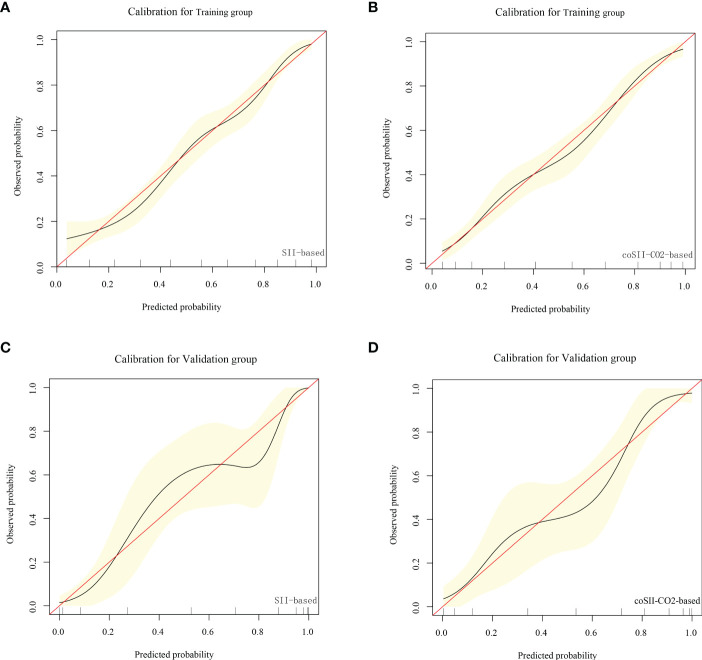
Calibration curve based on SII and coSII-CO_2_ in the training and validation groups. **(A, B)** Clinical calibration curve analysis of the training group. **(C, D)** Clinical calibration curve analysis of the validation group.

To further predict the GOS of patients with severe TBI at 6 months after discharge, we used the logistic regression model (age, SBP, pupil size, SAH, SII, PLR, [K^+^], [Ca^2+^], INR, CRP and coSII-CO_2_) to construct nomograms (**Figure 4**). In the training group, the C-index (age, SBP, pupil size, SAH, SII, PLR, [K^+^], [Ca^2+^], INR and CRP) based on the SII nomogram was 0.837. The C index (age, SBP, pupil size, SAH, PLR, [K^+^], [Ca^2+^], INR, CRP and coSII-CO_2_) of the coSIi-CO_2_-based nomogram was 0.860. In the external validation group, the C-index (age, SBP, pupil size, SAH, SII, PLR, [K^+^], [Ca^2+^], INR and CRP) based on the SII nomogram was 0.907. The C index (age, SBP, pupil size, SAH, PLR, [K^+^], [Ca^2+^], INR, CRP and coSII-CO_2_) based on the coSII-CO_2_ nomogram was 0.916 (**Figure 5**). DCA also confirmed that the coSII-CO_2_-based nomogram had a higher clinical net benefit for GOS at 6 months after discharge in most cases than the SII-based nomogram, in both the training and external validation groups (**Figure 6**). In addition, the calibration curve based on the coSII-CO_2_ nomogram showed better agreement between the predicted GOS score and the actual observed results at 6 months after discharge than the calibration curve based on the SII nomogram (**Figure 7**).

### Comparison of coSII-CO2 models with conventionally based CRASH and IMPACT models

**Figure 8:**
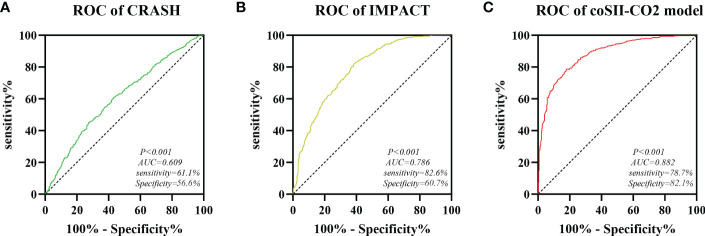
Comparison of predictive performance between CRASH, IMPACT and coSII-CO2 models. **(A)** The AUC value of CRASH is 0.609. **(B)** The AUC value of IMPACT is 0.786. **(C)** The AUC value of coSII-CO2 is 0.882.

CRASH (corticosteroid randomisation after significant head injury) model and IMPACT (international mission for prognosis and prognosis clinical trial (TBI) model is a commonly used and recognized prognostic model for traumatic brain injury. Based on our data study, we compared our coSII-CO2 model with the basic CRASH and IMPACT models. The AUC values of CRASH, IMPACT and coSII-CO2 models were 0.609, 0.786 and 0.882, respectively. The conclusion is that the accuracy of our prediction model in severe craniocerebral trauma patients is higher than that of the basic CRASH and IMPACT models (**Figure 8**).

## Discussion

In this study, we evaluated the prognostic effects of inflammatory indicators at admission (SII, PLR, NLR and LMR) and the peripheral blood CO_2_ concentrations in patients with severe TBI, compared the prognostic accuracy of these indicators, established corresponding nomograms, and tested their predictive performance. These results may help clinicians intuitively assess individual survival outcomes in treatment and guide clinical practice. The accuracy ranking of the area under the ROC curves were as follows: coSII-CO_2_: 0.751 > SII: 0.676 > CO_2_: 0.648 > PLR: 0.636 > LMR: 0.632 > NLR: 0.617. Patients with severe TBI with high levels of SII and low levels of CO_2_ at admission had poorer outcomes. In addition, coSII-CO_2_ and SII have been shown to be independent prognostic factors for GOS in patients with severe TBI.

Neuroinflammation, which develops rapidly after TBI, has become a research hotspot in recent years. It is characterized by the activation of resident cells, migration and recruitment of peripheral inflammatory cells, and release of inflammatory mediators (10, 28). Damage to brain tissue results in the release of many endogenous factors, such as RNA, DNA, heat shock proteins and high mobility group protein B1 (HMGB1), which act as damage-associated molecular patterns, bind Toll-like receptors, and consequently activate the NF-κB and MAPK pathways. A variety of inflammatory factors are released, including cytokines (IL-1β and IL-6), chemokines and immune receptors (29, 30). Subsequently, the kinin–kallikrein system, oxidative stress response, excitatory toxicity and the innate immune system are activated through a cascade of signals, thus leading to recruitment of neutrophils, mitochondrial dysfunction and polarization of microglia. In turn, downstream pathways are triggered, thereby leading to inflammatory responses in brain tissue, oxidative stress damage and the disruption of tight junctions. The activation of matrix metallopeptidase-9 (MMP-9) during this process exacerbates the TBI-induced disruption of BBB permeability (31). Neutrophils migrate rapidly from the damaged vasculature to the corresponding cortical and hippocampal parenchyma after TBI. During this process, the migration of neutrophils is closely associated with activated endothelial cells, and the interaction between activated neutrophils and endothelial cells plays an important role in secondary injury after TBI (32). In fact, neutrophils and other immune cells secrete factors including platelet activator, leukotriene B4 and IL-18 in an autocrine manner, thus amplifying their activation. The process is difficult to stop after the danger signal is no longer present, consequently, neutrophils indiscriminately cause brain tissue damage (33, 34). Other studies have shown that the infiltration of T lymphocytes, monocytes, B cells and microglia after polarization plays a complex role in TBI, causing different effects, such as repair or aggravation of brain injury, at different periods (11, 12, 35, 36). Therefore, the infiltration of inflammatory cells after TBI and downstream reactions play indispensable roles in TBI. In our study, patients with GOS score less than or equal to 8 at admission were included, and the relationship among inflammatory factors, CO_2_ concentration and prognosis in patients with severe and extremely severe craniocerebral injury was emphatically studied. Few articles have conducted detailed studies on such patients.

Several recent clinical studies have shown that inflammatory indicators after TBI, such as NLR and PLR, play important roles in predicting the clinical outcomes of neurotraumatic diseases (37–41). For example, a study by Chen et al. based on 688 cases of severe head trauma showed that 508 cases (73.8%) of patients with poor prognosis 1 year after head trauma. The NLR value of the poor outcome group at admission was significantly higher than that of the good outcome group. Multivariate logistic analysis showed that the higher NLR was correlated with the adverse outcome. The NLR value of the patients at admission increased the poor prognosis and functional outcome and mortality of the patients with severe traumatic brain injury within 1 year. NLR may serve as an readily available clinical marker for preoperative prognosis in patients with severe TBI, and high NLR on admission is associated with poor prognosis in patients with neurological injury (16). Paradoxically, however a single-center study of 255 patients showed that routine blood tests including (NLR) measured at admission were not significant predictors of sTBI outcomes (42). In our study, NLR univariate analysis was significant, but after multivariate analysis, NLR was not an independent risk factor. It has been reported that SII is associated with the prognosis of patients with various tumors, such as gastric cancer, bladder cancer and rectal cancer (43–45). Some studies have also suggested that the higher the SII, the worse the prognosis of patients with cerebral hemorrhage and cerebral venous thrombosis (CVT) (46). A study of 95 patients with traumatic intracerebral hemorrhage (TICH) showed that NLR and SII were significantly correlated with GCS scores and were promising predictors of clinical outcomes in patients with TICH (47). But so far, there are no studies on the predictive effect of SII in patients with severe craniocerebral trauma. In addition, there are many emerging TBI biomarkers in the study of predicting the prognosis of TBI, for example, protein biomarkers for neuronal cell body injury (UCH-L1, NSE), astroglial injury (GFAP, S100B), neuronal cell death (alpha II-spectrin breakdown products), axonal injury (NF proteins), white matter injury (MBP), post-injury neurodegeneration (total Tau and phospho-Tau), The post-injury autoimmune response (brain antigen-targeting autoantibodies), however, these markers are still in the clinical trial stage, and not all hospitals have the ability to obtain these markers (48). In our study, SII, NLR, PLR, LMR and peripheral blood carbon dioxide concentration were used for the first time for analysis. The factors required by the study are indicators that can be detected in most hospitals, and the prediction model has good accuracy, which can be widely used in clinical practice. The prognosis of patients with severe craniocerebral injury is poor in clinic, so an accurate model is needed to predict the prognosis so as to guide our clinical work.

To our knowledge, high concentration of peripheral blood CO_2_ will inhibit the process of inflammation through some ways, and inflammatory factors will also affect the prognosis of patients. However, there is no report on the combination of the two. This is the first report on the clinical and prognostic value of SII combined with peripheral blood CO_2_ concentration in patients with TBI. In the study, we had an independent external validation group and divided the different indicators into normal and outliers to account for possible baseline inconsistencies across hospitals. In addition, in the multiple regression analysis (nomogram), different scores occupied by SII alone and SII combined with CO_2_ were clearly listed, and coSII-CO_2_ was grouped in detail for clinical reference. The results also showed that the SII combined with CO_2_ model had better predictive performance and better clinical benefit. First, we used ROC analysis to calculate the predictive accuracy of SII, CO_2_, PLR, NLR, LMR and coSII-CO_2_ for GOS 6 months after discharge. The AUC values were as follows: SII=0.676, CO_2_ = 0.648, PLR=0.636, NLR=0.617, LMR=0.623 and coSII-CO_2_ = 0.751. Compared with SII or CO_2_ alone, coSII-CO_2_ had the highest AUC. Patients with high SII and low CO_2_ had significantly poorer GOS at 6 months after discharge than patients with low SII and high CO_2_. Multivariate logistic regression analysis revealed that age, SBP, pupil size, SAH, SII, PLR, [K^+^], [Ca^2+^], INR, CRP and coSII-CO_2_ (P < 0.001) were independent prognostic factors affecting GOS in patients with severe TBI. Subsequently, 802 patients in our hospital were selected as the training group, and 207 patients in two other centers were selected as the external validation group to construct the corresponding nomograms. In the training group, the C-index based on the SII nomogram was 0.837. The C-index of the coSII-CO_2_ based nomogram was 0.860. In the external validation group, the C-index based on the SII nomogram was 0.907. The C-index of the coSII-CO_2_ based nomogram was 0.916. In addition, our DCA confirmed that coSII-CO_2_-based nomograms had a net clinical benefit superior to that of SII-based nomograms at 6 months after discharge in most cases. Furthermore, the coSII-CO_2_-based calibration curve for the probability of GOS at 6 months after discharge showed better agreement with the actual observed results than the calibration curve based on the SII nomogram. These results supported our hypothesis that the combination of SII and CO_2_ improves prognostic accuracy for patients with TBI.

In summary, exploration of the mechanism of inflammatory response after TBI is important for applications in patient treatment and prognostication. The SII, which combines neutrophils, lymphocytes and platelets, and reflects inflammation and poor prognosis in patients with TBI, is more reliable and representative than NLR, LMR and PLR. In our study, SII and coSII-CO_2_ were independent predictors of 6-month adverse outcomes in patients with TBI.

Despite demonstrating the prognostic value of SII and coSII-CO_2_ in patients with TBI, this study has several limitations. First, this was a retrospective analysis; therefore, several factors might have influenced the results of the study. Second, although this was a multi-center study, the number of patients was not sufficient, and this study calculated only the SII and CO_2_ at admission, without dynamic monitoring, which might have provided more reliable results. Finally, prospective studies are needed to confirm our conclusions.

## Conclusions

In conclusion, our study shows that SII and CO_2_ have better predictive performance than NLR, PLR and LMR. SII and CO_2_ can be used as new, accurate and objective clinical predictors, and coSII-CO_2_, based on combining SII with CO_2_, can be used to improve the accuracy of GOS prediction in patients with TBI 6 months after discharge.

## Data availability statement

The original contributions presented in the study are included in the article/Supplementary Material. Further inquiries can be directed to the corresponding authors.

## Ethics statement

The studies involving human participants were reviewed and approved by the ethics committee of the 900th Hospital. Written informed consent for participation was not required for this study in accordance with the national legislation and the institutional requirements.

## Author contributions

Conceptualization, LC. Data curation, LC, TF, and SX. Formal analysis, LC, YZ and SX. Funding acquisition, XX and SW. Methodology, LC, YL and SW. Project administration, XX and SW. Resources, LC and SX. Writing original draft, LC, QC and XQ. Writing, review and editing, XX and SW. All authors have read and agreed to the published version of the manuscript.
